# Prolonged Aβ treatment leads to impairment in the ability of primary cortical neurons to maintain K^+ ^and Ca^2+ ^homeostasis

**DOI:** 10.1186/1750-1326-5-30

**Published:** 2010-08-13

**Authors:** Lana Shabala, Claire Howells, Adrian K West, Roger S Chung

**Affiliations:** 1 NeuroRepair Group, Menzies Research Institute, University of Tasmania. Private Bag 23, Hobart, Tasmania, 7001, Australia

## Abstract

**Background:**

Alzheimer's disease (AD) is a progressive neurodegenerative disease, characterised by the formation of insoluble amyloidogenic plaques and neurofibrillary tangles. Beta amyloid (Aβ) peptide is one of the main constituents in Aβ plaques, and is thought to be a primary causative agent in AD. Neurons are likely to be exposed to chronic, sublethal doses of Aβ over an extended time during the pathogenesis of AD, however most studies published to date using *in vitro *models have focussed on acute studies. To experimentally model the progressive pathogenesis of AD, we exposed primary cortical neurons daily to 1 μM of Aβ_1-40 _over 7 days and compared their survival with age-similar untreated cells. We also investigated whether chronic Aβ exposure affects neuronal susceptibility to the subsequent acute excitotoxicity induced by 10 μM glutamate and assessed how Ca^2+ ^and K^+ ^homeostasis were affected by either treatment.

**Results:**

We show that continuous exposure to 1 μM Aβ_1-40 _for seven days decreased survival of cultured cortical neurons by 20%. This decrease in survival correlated with increased K^+ ^efflux from the cells. One day treatment with 1 μM Aβ followed by glutamate led to a substantially higher K^+ ^efflux than in the age-similar untreated control. This difference further increased with the duration of the treatment. K^+ ^efflux also remained higher in Aβ treated cells 20 min after glutamate application leading to 2.8-fold higher total K^+ ^effluxed from the cells compared to controls. Ca^2+ ^uptake was significantly higher only after prolonged Aβ treatment with 2.5-fold increase in total Ca^2+ ^uptake over 20 min post glutamate application after six days of Aβ treatment or longer (P < 0.05).

**Conclusions:**

Our data suggest that long term exposure to Aβ is detrimental because it reduces the ability of cortical neurons to maintain K^+ ^and Ca^2+ ^homeostasis in response to glutamate challenge, a response that might underlie the early symptoms of AD. The observed inability to maintain K^+ ^homeostasis might furthermore be useful in future studies as an early indicator of pathological changes in response to Aβ.

## Background

Alzheimer's disease (AD) is the most common form of dementia within the ageing population and accounts for between 50% and 60% of dementia cases [1]. Sufferers of AD experience progressive loss of memory and cognitive abilities that eventually lead to dementia and death. The pathological hallmarks of the disease include extracellular β-amyloid (Aβ) plaques, intracellular neurofibrillary tangles (NFTs) and dystrophic neurites (DNs) [2]. The Aβ peptide is one of the main constituents in Aβ plaques, and is thought to be a primary causative agent in AD, significantly contributing towards AD pathogenesis.

AD is a progressive disease which develops over many years, even decades. Therefore the pathogenesis of AD does not entail a sudden insult of Aβ which causes widespread neuronal death within the brain. Instead, there appears to be a gradual progression of the disease which involves the accumulation of soluble Aβ within the brain due to the chronic imbalance between production and clearance of Aβ. This gradual accumulation of Aβ over extended periods of time leads to the formation of the insoluble Aβ aggregates which form the characteristic plaques, but it also modifies neuronal function. It is difficult to correlate early stages of AD pathogenesis with the accumulation of Aβ, as patients remain asymptomatic. Studies using transgenic mice which express human mutant Aβ precursor peptide (APP, Tg2576 mice) demonstrated that the increase of Aβ_1-40 _and Aβ_1-42 _over several months was accompanied by deficits in normal learning and memory [3,4]. When the Aβ load was minimal, mice demonstrated normal spatial learning and memory, indicating that the gradual build up of Aβ correlated with the physiological changes associated with AD. Studies have demonstrated that many molecular forms of Aβ are neurotoxic causing neuronal cell death *in vitro *and neuronal loss *in vivo*. For example, synthetic Aβ peptides were toxic to hippocampal and cortical neurons in culture [5,6].

Neurons are likely to be exposed to sublethal doses of Aβ over an extended time during the pathogenesis of AD. However studies to date have not developed an experimental model of this chronic exposure. There have been numerous *in vitro *models used to examine Aβ neurotoxicity using various forms and concentration of the peptide, over the short term. For instance Deshpande and colleagues examined the toxicity of a single dose of 5 μM fibrillar Aβ_1-42 _and Aβ-derived diffusible ligands on human cortical neuron cultures for up to 24 hours [6]. At the same time studies examining the chronic exposure of neurons to Aβ at sublethal doses would provide valuable information relative to the physiological processes which occur in the AD brain.

The accumulation of toxic Aβ peptide aggregates in AD brain is thought to trigger the extensive synaptic loss and neurodegeneration linked to cognitive decline, an idea that underlies the 'amyloid hypothesis' of AD etiology in both the familial and sporadic forms of the disease [7]. Recent reports strongly suggest that in the initial stages of AD, glutamate receptors are dysregulated by Aβ accumulation resulting in disruption of glutamatergic synaptic transmission which parallels early cognitive deficits [8]. High concentrations of glutamate have been documented to cause neuronal degeneration in various *in vivo *and *in vitro *models [9,10]. Therefore, it would be of interest whether chronic neuron exposure to Aβ would sensitise them to excitotoxicity caused by increased glutamate concentration.

Excessive glutamatergic stimulation is associated with an increase in intracellular calcium ([Ca^2+^]_i_). Although Ca^2+ ^is necessary for a number of physiological processes, excessive amounts may lead to neuronal dysfunction and cell death. Neuronal increases in [Ca^2+^]_i _can activate a number of enzymes, such as phospholipases, proteases, endonucleases and nitric oxide synthase (NOS) that are associated with neuronal cell death [11]. Increases in cytosolic Ca^2+ ^levels were observed in AD mouse models strongly supporting the theory that Ca^2+ ^dysregulation is involved in AD [12-14]. Recent data also indicate that rodent and human Aβ modulate K^+ ^currents [15]. Voltage-dependent K^+ ^(Kv) channels act as potent modulators of diverse excitatory events that are linked to glutamatergic neurotransmission [16] including modulation of Ca^2+ ^uptake. Changes in ion fluxes are one of the earliest events in neural responses to Aβ treatment that precede neuron survival. Therefore, delineation between neuronal ability to maintain Ca^2+ ^and K^+ ^homeostasis during prolonged Aβ treatment and their viability might provide inside into mechanisms underlying processes of neurodegeneration.

To experimentally model the progressive pathogenesis of AD, primary cortical neurons were exposed to sublethal doses of Aβ_1-40 _over an extended period of time of 7 days. In this study we compared survival of cortical neurons treated for different periods of time with Aβ_1-40 _and correlated them to the neuron's ability to maintain homeostasis of Ca^2+ ^and K^+ ^ions under the same conditions. Finally, we investigated whether prolonged exposure to relatively low Aβ_1-40 _(1 μM) increased neuronal susceptibility to subsequent acute excitotoxicity induced by the neurotransmitter glutamate.

## Results

### Continuous exposure to Ab_1-40 _is mildly neurotoxic to cultured cortical neurons

**Figure 1:**
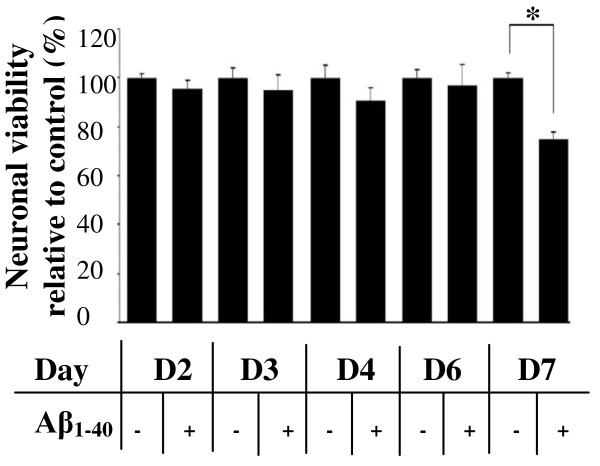
**Viability of cortical neurons in response to daily treatments of Aβ_1-40_**. 6DIV cortical neurons were treated daily with 1 μM Aβ_1-40 _for seven days. Neuronal viability was recorded at Day 2, 3, 4, 6 and 7 of treatment and expressed as the percentage of surviving Aβ-treated neurons compared to age-matched, untreated controls. At Day 7, neuronal viability was significantly lower than the vehicle-treated controls. *P < 0.05 between Aβ_1-40 _treated and age-similar control on the same day of measurement. All data are means ± standard error of the mean (SEM), n = 4.

We first examined whether chronic exposure of mature neurons to low levels of Aβ caused cell death. Cortical neurons were maintained for 6 DIV, by which time they had formed a dense meshwork of neuritic processes. They were then treated with soluble monomeric Aβ_1-40 _(1 μM) daily for up to a further seven days. Daily determination of neuronal viability by an Alamar Blue assay revealed that Aβ_1-40 _treatment did not reduce cell viability for cells treated for up to six days (Figure 1). However, by seventh day of the treatment neuronal viability decreased by 20% (P < 0.05).

### Acute exposure to low doses of Aβ_1-40 _does not change K^+ ^and Ca^2+ ^fluxes from neurons

Inability to maintain Ca^2+ ^homeostasis has been strongly implicated in AD [12-14]. Therefore, we investigated whether ion homeostasis would be affected by Aβ. We first examined whether acute exposure of mature neurons to low levels of a soluble Aβ_1-40 _affect changes in K^+ ^and Ca^2+ ^fluxes. We therefore performed simultaneous recordings of K^+ ^and Ca^2+ ^fluxes from cortical neurons using Microelectrode Ion Flux Estimation (MIFE) after an acute application of 1 μMAβ_1-40 _to primary 14 DIV neurons (Additional file 1, Figure S1). MIFE enables sensitive, real time measurement of ion fluxes across cell membranes in a variety of cell models and is non-invasive [17,18]. No changes in net K^+ ^and Ca^2+ ^fluxes were observed within 15 min post treatment with 1 μM Aβ_1-40 _that served as a control for our further experiments. We also found no changes in net K^+ ^and Ca^2+ ^fluxes in response to vehicle (Additional file 2, Figure S2) further confirming our findings.

### Continuous exposure to Aβ_1-40 _increases K^+ ^efflux from neurons

**Figure 2:**
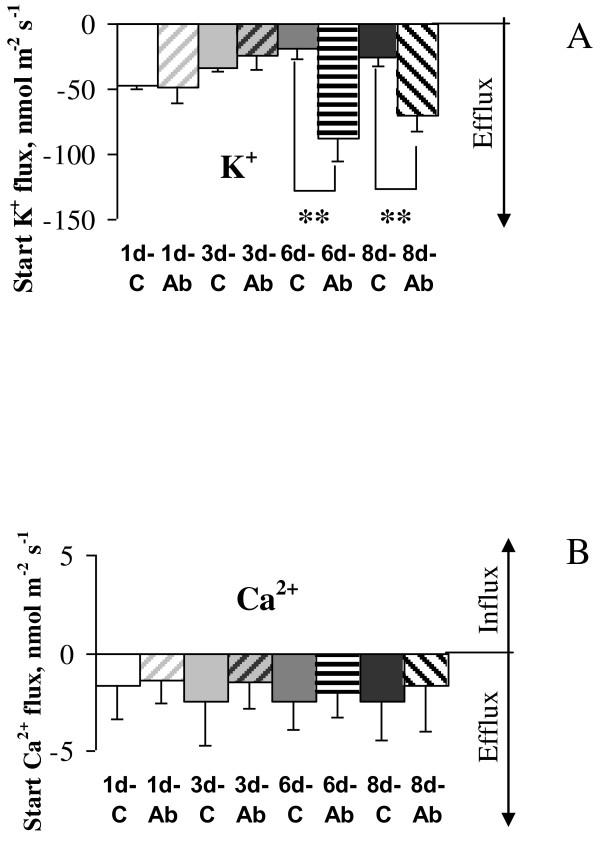
**Effect of daily treatment with Aβ_1-40 _on K^+ ^and Ca^2+ ^fluxes**. After 6DIV, cortical neurons were treated daily with 1 μM Aβ_1-40 _for up to eight days. Net ion fluxes were recorded daily from monolayers of cells grown on glass cover slips treated with poly-L-lysine for cell adherence. Data was acquired non-invasively using the microelectrode MIFE technique. Steady-state fluxes of K^+ ^(**A**) and Ca^2+ ^(**B**) are shown for one, three, six, and eight day treatment with Aβ_1-40_. The sign convention is 'influx positive'. While K^+ ^flux remained similar in control and cells treated with Aβ for one and three days, an increase in duration of the treatment to six days or longer led to a four-fold increase in K^+ ^efflux from the neural cells (**P < 0.02). No difference in the levels of Ca^2+ ^fluxes between control and Aβ treated cells was observed. Error bars are SEM (n = 4-7).

Under physiological conditions neurons are continuously exposed to low doses of Aβ. To simulate those conditions we treated neurons with 1 μM Aβ over a prolonged period of time measuring ion fluxes of interest. Therefore, we investigated whether chronic treatment with soluble Aβ_1-40 _can affect the ability of cortical neurons to maintain Ca^2+ ^homeostasis (Figure 2). We also tested whether K^+ ^homeostasis would be affected by Aβ treatments since fluxes of this ion are pivotal to neuronal activity. No difference was observed in the level of Ca^2+ ^fluxes between untreated cells, and cells treated with Aβ_1-40 _for up to eight days (Figure 2A). At the same time significant differences were observed in K^+ ^fluxes (Figure 2B). While the K^+ ^flux was similar in control cultures and those treated with Aβ_1-40 _for one and three days, treatment with a soluble monomeric Aβ_1-40 _(1 μM) daily for a period of six days or longer led to a significantly higher K^+ ^efflux from the neural cells. Specifically, six and eight day Aβ treatment resulted in four- and three-fold increase in K^+ ^efflux from the cells, respectively (P < 0.02)suggesting that chronic treatment with Aβ_1-40 _leads to a reduced capacity of neurons to maintain K^+ ^homeostasis.

### Chronic exposure to Aβ_1-40 _does not decrease neuronal viability following excitotoxic stress but does affect subsequent K^+ ^homeostasis

**Figure 3:**
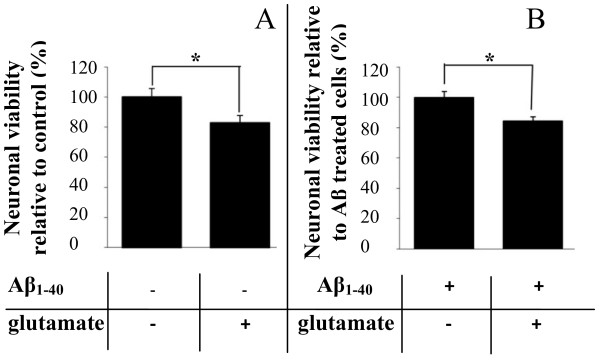
**Neuronal viability in response to glutamate treatment following seven days of daily Aβ_1-40 _treatment**. Treatment with glutamate significantly reduced neuronal viability of cultured cortical neurons by approximately 20% compared to untreated cells (**A**). When neurons were pre-treated with 1 μM Aβ_1-40 _for seven days, challenge with 200 μM glutamate for 24 hours resulted in a comparable degree of neuronal death compared to unchallenged, Aβ_1-40 _-treated cells (**B**). *P < 0.05 between treatments. All data are means ± SEM, n = 6.

**Figure 4:**
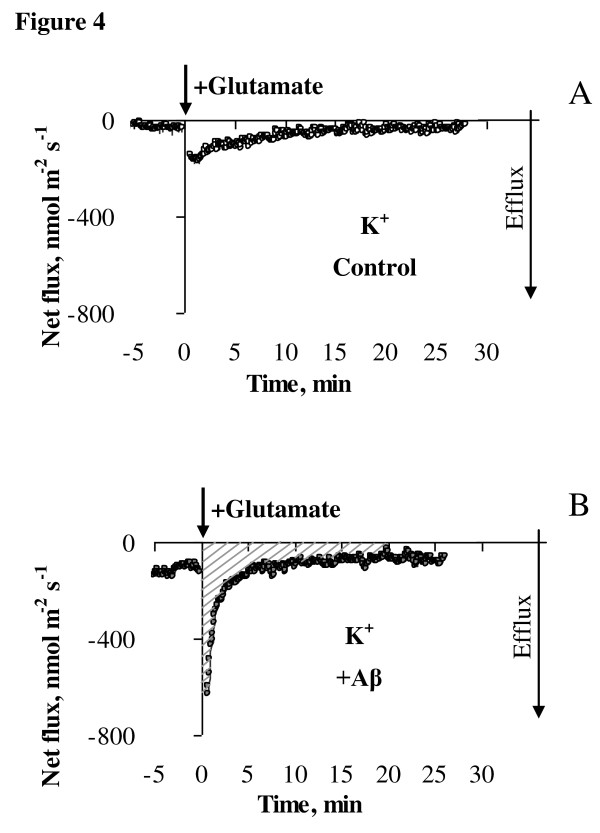
**Kinetics of K^+ ^flux in response to acute application of 10 μM glutamate**. K^+ ^flux was measured from age-similar control cells (**A**) and primary cortical neurons treated daily with 1 μM Aβ_1-40 _for six days (**B**). Flux values were recorded for 5 min before glutamate application (-5 to 0 min) and 25 min afterwards with data acquired at a rate of 10 samples/sec and averaged over every 6 sec. Glutamate (10 μM) was applied at zero time as indicated by an arrow and led to a transient K^+ ^efflux from neurons that returned to pre-stress conditions within 15 min afterwards. Notably, the magnitude of K^+ ^efflux was higher in Aβ treated cells as shown by comparison of the peak values. Negative values of K^+ ^flux indicate "efflux". Shaded area in panel **B **indicates total K^+ ^efflux over 20 min after glutamate treatment. One (out of 7) typical example is shown.

**Figure 5:**
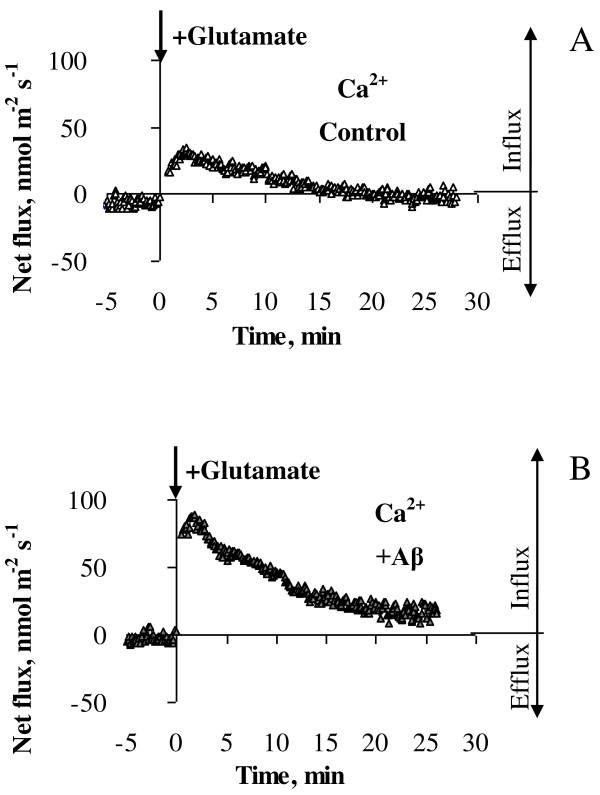
**Kinetics of Ca^2+ ^fluxes in response to acute application of 10 μM glutamate**. Ca^2+ ^fluxes were measured from age-similar control cells (**A**) and primary cortical neurons treated daily with 1 μM Aβ_1-40 _for six days (**B**). Flux values were recorded for 5 min before glutamate application (-5 to 0 min) and 25 min afterwards with data acquired at a rate of 10 samples/sec and averaged over every 6 sec. Glutamate (10 μM) was applied at zero time as indicated by an arrow and led to Ca^2+ ^influx into cultured cortical neurons (positive flux values). One (out of 7) typical example is shown.

We next investigated whether neurons exposed to prolonged Aβ_1-40 _treatment had increased susceptibility to subsequent excitotoxicity induced by 200 μM glutamate. While glutamate treatment for 24 hours killed approximately 20% of neurons in the absence of any Aβ_1-40 _treatment (Figure 3A), we found that seven days of pre-treatment with Aβ_1-40 _did not further elevate neuronal susceptibility to glutamate-induced neurotoxicity (Figure 3B). We then examined K^+ ^and Ca^2+ ^fluxes in neurons which were treated with 1 μM Aβ_1-40 _daily for a period from one to eight days and then challenged with 10 μM of glutamate. Using MIFE, net fluxes of K^+ ^and Ca^2+ ^were monitored continuously throughout the experiment starting before the glutamate challenge and up to 30 min afterwards with data acquired every 6 sec. Typical examples of kinetics of net K^+ ^and Ca^2+ ^fluxes in response to challenge with glutamate are shown in Figures 4A-B and 5A-B, respectively.

**Figure 6:**
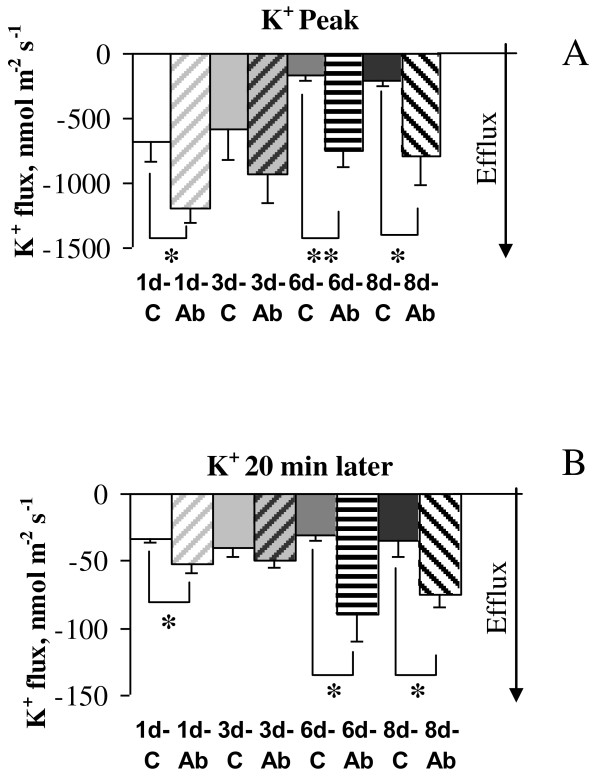
**Effect of glutamate application on K^+ ^fluxes**. Glutamate (10 μM) was applied to cortical neurons following daily treatment with 1 μM Aβ_1-40_, and to age-matched un-treated control cells. Peak values of K^+ ^fluxes (**A**) and steady-state K^+ ^fluxes recorded 20 min after glutamate application (**B**) are shown for one, three, six, and eight days of treatment with Aβ. Peak K^+ ^efflux was substantially (1.6-fold) higher after one day of treatment with soluble Aβ_1-40 _than in age-similar control cells and further increased to 5.7-fold difference after six days of treatment. The capacity of cortical neurons to retain K^+ ^flux at pre-stress levels after glutamate challenge was assessed by comparing steady-state values of K^+ ^fluxes 20 min post-treatment. K^+ ^efflux in Aβ treated cells was significantly higher than in age-similar controls after treatment with Aβ suggesting that Aβ accumulation by neurons reduces their ability to maintain K^+ ^homeostasis. * - P < 0.05, ** - P < 0.02, t-test. Error bars represent SEM (n = 4-7).

Acute treatment of cortical neurons with 10 μM glutamate led to a dramatic K^+ ^efflux from neurons that returned to pre-stress conditions within 20 min after the challenge (Figures 4A, B). Notably, the magnitude of K^+ ^efflux was higher in cells treated with Aβ for six days or longer (peak values in the graphs). We therefore compared the magnitudes of the peak K^+ ^efflux from neurons treated with Aβ with age-similar controls and found that K^+ ^efflux was substantially (1.6-fold) higher even after one day of treatment with soluble Aβ_1-40 _(Figure 6A). This difference was more pronounced with increase of duration of the treatment with Aβ leading to more than 3.5-fold increase in the peak K^+ ^efflux after six and eight days of treatment with soluble monomeric Aβ_1-40 _(1 μM), (Figure 6A).

We also assessed capacity of the cortical neurons to return K^+ ^flux to pre-stress levels after glutamate challenge. For this we compared steady-state values of K^+ ^fluxes 20 min after glutamate challenge in Aβ treated and age-similar un-treated control neurons (Figure 6B). While the level of K^+ ^flux had returned to basal levels in non Aβ treated cells, this was not the case when neurons had received continual treatment with Aβ, suggesting that Aβ_1-40 _treated neurons have reduced capacity to maintain K^+ ^homeostasis in response to glutamate stimulation. We also calculated total K^+ ^efflux over 20 min after acute glutamate application shown as shaded area in Figure 4B. Total amount of K^+ ^efflluxed from the cells over 20 min post glutamate application was substantially higher after treatment with low concentration of Aβ for six days or longer. For example, it was 2.8-fold higher in cells pre-treated with Aβ for six days than in age-similar controls (22228.1 ± 2800.13 vs 8033.46 ± 3799.76 nmol m^-2^, respectively, P < 0.05) further suggesting dysregulation of K^+ ^homeostasis in Aβ treated cells.

### Chronic Aβ treatment leads to increased Ca^2+^uptake by cortical neurons in response to glutamate challenge

**Figure 7:**
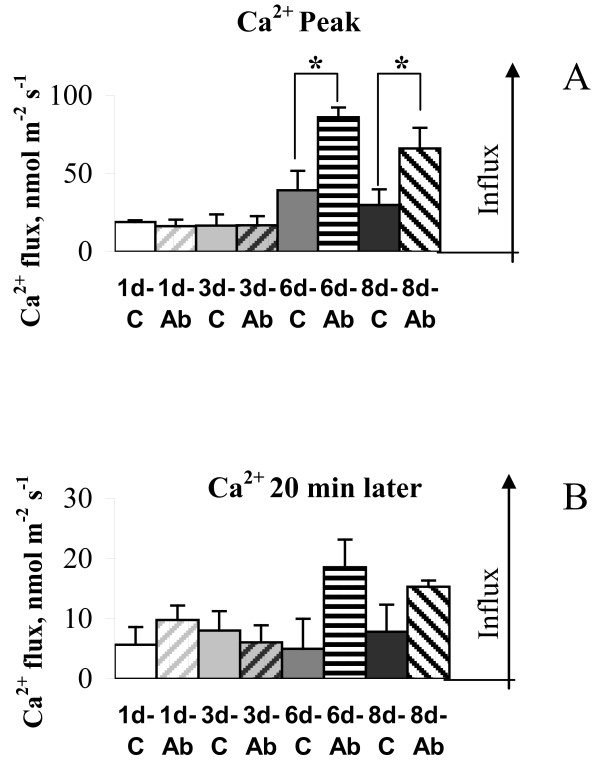
**Effect of glutamate application on Ca^2+ ^fluxes**. Glutamate (10 μM) was applied to cortical neurons following daily treatment with 1 μM Aβ_1-40_, and to age-matched un-treated control cells. Peak values of Ca^2+ ^fluxes (**A**) and steady-state Ca^2+ ^fluxes recorded 20 min after glutamate application (**B**) are shown for one, three, six, and eight days of treatment with Aβ. Glutamate application to the bath induced a mild Ca^2+ ^influx in primary cortical neurons with peak values being not statistically different from the relevant Ca^2+ ^peak values recorded from age-similar control cells one and three days after the treatment. However, treatment with soluble Aβ for six days and longer increased Ca^2+ ^uptake by more than two-fold as compared with non-treated age-similar control cells (* - P < 0.05, t-test). While steady-state values of Ca^2+ ^fluxes measured 20 min after the glutamate challenge were not statistically different from the relevant values of age-similar control cells even after six days of Aβ treatment (Figure 7B; P < 2.0 at n = 7), the calculated total amount of Ca^2+ ^taken up by Aβ treated cells over 20 minutes of treatment with glutamate was 2.5-fold higher than in age-similar un-treated control cells (P < 0.05). Error bars represent SEM (n = 4-7).

We similarly used MIFE to examine Ca^2+ ^fluxes following glutamate challenge to control and Aβ treated cortical neurons. Neurons which had been exposed to glutamate challenge (10 μM) rapidly took up Ca^2+ ^followed by a return to pre-stress levels (Figure 5A, B). In neurons which had been exposed to Aβ for one and three days, glutamate induced an influx of Ca^2+ ^that was not statistically different from age-similar control cells (Figure 7A). However, cortical neurons that received soluble Aβ_1-40 _for six consecutive days or longer had a significant impairment in their ability to maintain Ca^2+ ^homeostasis, resulting in significantly greater Ca^2+ ^uptake following excitotoxic stimulation with glutamate (Figure 7A). The magnitude of the peak Ca^2+ ^influx was substantially higher in Aβ-treated cells than in the age-similar control cells (Figure 7A). At the same time steady-state values of Ca^2+ ^fluxes measured 20 min after the glutamate challenge were not statistically different from the relevant values of age-similar control cells even after six and eight days of Aβ treatment, despite an apparent trend towards an increase in Ca^2+ ^flux values (Figure 7B). However, a calculation of total Ca^2+ ^uptake over 20 min of treatment with glutamate, showed that the amount of Ca^2+ ^taken up by Aβ treated cells was 2.5-fold higher than in age-similar un-treated control cells after six days of treatment with Aβ (7964.47 ± 672.84 vs 3156.41 ± 1615.93 nmol m^-2^, respectively, P < 0.05) further confirming Ca^2+ ^dysregulation in cortical neurons after prolonged exposure to a soluble Aβ.

## Discussion

### Chronic exposure to Ab_1-40 _is neurotoxic to cultured cortical neurons

The accumulation of Aβ protein in the brain is considered to be a key factor that causes AD. Most cultured cell models have examined the acute toxicity of Aβ, typically following 24 h treatment [6] which may not reflect the situation in vivo. In this study we examined the effect of daily exposure to moderate levels of soluble, monomeric Aβ_1-40 _(1 μM) for up to seven days and demonstrated, for the first time, that chronic exposure of primary cortical neurons to levels of Aβ which have no evident effect on viability over the short term, are indeed neurotoxic. A decrease in cell survival was only observed after seven days of exposure to 1 μM Aβ, with a 20% decrease in cell viability. This finding indicates that Aβ_1-40 _accumulation over time can induce neurodegenerative changes to neurons and thus contribute to the slowly progressing pathogenesis of AD.

### Chronic exposure to Aβ_1-40 _impairs K^+ ^homeostasis in cortical neurons

Using MIFE, we observed an increase in K^+ ^efflux concurrent with the decline in neuronal viability. While acute treatment with 1 μM Aβ did not affect net fluxes of K^+ ^nor Ca^2+ ^(Additional file 1: Figure S1) confirming earlier findings regarding intracellular Ca^2+ ^changes [19], here we demonstrate that prolonged treatments with low concentrations of Aβ_1-40 _led to dysregulations of K^+ ^but not Ca^2+ ^homeostasis. This is in agreement with an earlier demonstration that exposure of neurons to Aβ peptide can enhance voltage gated K^+ ^currents [20-22]. For example, preincubation of cells with recombinant human or rat Aβ_1-40 _was shown to significantly increase K^+ ^channel current density and levels for Kv4.2 [15]. Studies on brain slices at different stages of AD demonstrated that voltage gated K^+ ^currents were already overexpressed in early stages of AD, and in advanced AD stages Kv3.4 was present at high levels in neurodegenerative structures [23]. Other studies also demonstrated effects of Aβ on activity of Na^+^/K^+^-ATPase. For instance, it was shown that exposure of cultured neurons to Aβ_1-40 _caused selective reduction in Na^+^/K^+^-ATPase activity which preceded loss of Ca^2+ ^homeostasis and cell degeneration [24]. This was further confirmed in studies using APP and PS1 deficient mice [25]. Together, enhancements of voltage gated K^+ ^channels and decrease in Na^+^/K^+^-ATPase activity would increase overall K^+ ^efflux, similar to Aβ effects observed in our experiments. The decrease in intracellular K^+ ^concentration is also consistent with a decrease in cell viability since this is required for apoptosome formation and activation of caspases and endonucleases during apoptosis [26,27].

### Exposure to glutamate further decreased impaired neuronal ability to maintain K^+ ^homeostasis in cells chronically treated with Aβ_1-40_

A growing body of evidence suggests that perturbations in systems using the excitatory amino acid L-glutamate may underlie chronic neurodegenerative disorders such as AD. Therefore, we investigated whether prolonged exposure to 1 μM Aβ_1-40 _increased neuronal susceptibility to subsequent acute excitotoxicity induced by glutamate. Surprisingly, no further decrease in neuronal viability was observed when cortical neurons treated with Aβ for seven days were exposed to glutamate (Figure 3A, B). Likewise, an earlier study has shown that low concentrations of Aβ peptide induced early and prolonged activation of proapoptoic markers in neurons without resulting in subsequent cell death in culture [28] most likely due to additional protective mechanisms in place at glutamate concentrations used. However, despite the absence of an overt change in neuronal viability following the combined treatments, we demonstrated that exposure to Aβ perturbed the ability of neurons to maintain K^+ ^homeostasis following glutamate treatment. Treatments with 1 μM Aβ for one day were sufficient to lead to glutamate induced K^+ ^dyshomeostasis in cortical neurons (Figures 6A, B). Indeed, peak K^+ ^efflux after acute exposure to 10 μM glutamate was substantially higher in Aβ treated cells than in age-similar controls. This difference increased with time of Aβ exposure from 1.6 to 4.8-fold for one and six days of the treatment, respectively. Neurons were also unable to return K^+ ^to pre-stress level as shown for steady-state K^+ ^fluxes measured 20 minutes after the treatment with glutamate (Figure 6B). Another consequence of chronic pretreatment with Aβ was a higher level of K^+ ^efflux even before challenge with glutamate (shown in Figure 2 for initial K^+ ^values). Overall, this led to a 2.9-fold higher amount of K^+ ^efflux from cells treated with Aβ_1-40 _for six days or longer after glutamate application than from age-similar control cells over 20 min of glutamate exposure. Taken together, our data suggest that chronic Aβ treatment impairs the ability of cortical neurons to maintain K^+ ^homeostasis.

### Prolonged Aβ exposure increased Ca^2+^uptake by cortical neurons in response to acute glutamate treatment

Ca^2+ ^homeostasis undergoes subtle dysregulation during physiological ageing making neurons more vulnerable to additional stress which, in turn, can lead to neuronal degeneration. Recent evidence strongly supports the theory that dysregulation of intracellular Ca^2+ ^homeostasis underlies the development of AD [12-14]. Therefore, we investigated whether prolonged exposure to continuous Aβ_1-40 _treatment followed by glutamate challenge would affect Ca^2+ ^homeostasis in cortical neurons. Acute treatment with 10 μM glutamate after chronic pre-treatment with 1 μM Aβ_1-40 _in our experiments led to net Ca^2+ ^uptake from external environment (Figure 5B). An increase in glutamate concentration leads to activation of ionotropic and metabotropic receptors [29,30] and Ca^2+ ^influx through both glutamate-dependent pathways and voltage-dependent Ca^2+ ^channels [31-33]. While the observed Ca^2+ ^uptake in response to acute glutamate treatment was expected, in the current study we showed that the magnitude of Ca^2+ ^uptake was substantially increased in cells treated with 1 μM Aβ_1-40 _for six consecutive days or longer compared to un-treated control cells (Figure 7A). Calculated total Ca^2+ ^taken up by Aβ treated cells over 20 min post glutamate application was 2.5-fold higher than in age-similar untreated control cells (P < 0.05) suggesting dysregulation of Ca^2+ ^homeostasis in cortical neurons after prolonged exposure to soluble Aβ. Overall, our data showed that prolonged Aβ accumulation renders cortical neurons more vulnerable to excitotoxicity leading to their inability to maintain Ca^2+ ^homeostasis when glutamate concentration increases.

## Conclusions

In this study we developed an *in vitro *model to test the effects of prolonged Aβ treatment. We demonstrated for the first time that continuous exposure to low levels of Ab_1-40 _induced neurodegeneration of cultured cortical neurons, with decreased viability evident after seven days of treatment. This decrease in viability correlated with increased neuronal K^+ ^efflux. We also demonstrated that long term exposure to Aβ_1-40 _leads to imbalance in K^+ ^and Ca^2+ ^homeostasis and contributes to glutamate-induced vulnerability and cell death at later time point. This suggests that chronic accumulation of low levels of Aβ_1-40 _may contribute to the slowly progressing pathogenesis of AD. The results also suggest that the inability to maintain ion homeostasis is an early indicator of cell vulnerability that precedes cell death and might be used as a sensitive tool to cell susceptibility to the excytotoxic treatment.

## Methods

### Rodent cortical neuron cultures

Cortical tissue was removed from embryonic day 18 Hooded Wistar rat embryos and incubated in sterile 10 mM HEPES buffer (37°C; Sigma). The cortical tissue was trypsinised (0.25%; Sigma) and washed with fresh HEPES buffer. The cell pellet was gently triturated and filtered through 60 μm gauze. Cells were then plated onto glass coverslips (254 mm^2^) pre-coated overnight with poly-L-lysine (Sigma) at the density of 1 × 10^5 ^cells/well and culture medium added to the wells. The culture medium consisted of Neurobasal™ medium (Gibco), supplemented with 10% FCS (Gibco), 2% (f/c) B-27 supplement (Gibco), 5 mM (f/c) L-glutamine (Sigma), and 10 mg/ml gentamycin (Sigma). Cultures were maintained at 37°C in humidified air containing 5% CO2. The culture medium was replaced with serum-free culture medium after 24 hours, followed by a full media change three times a week.

### Aβ treatment and toxicity assay

1 μM Aβ_1-40 _was applied to 6 DIV cortical neuron cultures daily for 7 days. Fresh media was applied a week. A biological reducing agent, 300 μM ascorbate (the physiological concentration within the brain), was freshly applied with each media change for both control and the treatment. At days 2, 3, 4, 6, and 7 the neuronal viability was measured using an Alamar Blue assay, which measures cellular metabolic activity [34]. The degree of cellular metabolic reduction of Alamar Blue was determined by fluorescence (excitation 535 nm, emission 595 nm), and expressed as the percentage of the signal obtained from the vehicle-treated culture. Day zero was the first day of treatment

### Neural survival following chronic Aβ treatment

To examine whether Aβ treatment increased neuron susceptibility to excitotoxicity, 6 DIV cultures treated daily with 1 μM of soluble Aβ_1-40 _for seven days were exposed to 200 μM glutamate. Glutamate was prepared as we have reported previously [35]. Briefly, D-glutamic acid was dissolved in appropriate medium (Neurobasal medium for viability assay and aCSF for MIFE experiments). Neuronal viability was measured using the Alamar Blue assay after 24 hours of glutamate treatment.

### Ion-selective flux measurements

The technique of Microelectrode ion flux estimation (MIFE) has been reviewed recently [18] and the complete experimental procedure including ion-selective microelectrode fabrication and cell preparation and immobilisation are given elsewhere [17,18]. Cortical neurons for the MIFE measurements were grown for 6 days at 1 × 10^5 ^cells/well on coverslips coated with poly-L-lysine as described above. By 6 DIV a dense monolayer of neurons had developed. Cells were washed in artificial CSF (aCSF) by dipping a coverslip with cells into a beaker with pre-warmed to 37°C aCMF. The coverslip then was placed into a measuring chamber with 2 mL of aCMF at 37°C, and left for adaptation for a further 40 min at room temperature (RT, 23°C) prior to an experiment. The composition of the aCSF was: 150 mM NaCl, 0.5 mM KCl, 0.5 mM CaCl_2_, 1.5 mM MgCl_2_, 1.25 mM NaH_2_PO_4_, 2 mM NaHCO_3_, 25 mM glucose, pH 7.2. Measurements were taken after one, three, six, and eight days post treatment with Aβ_1-40_. Control experiments used age similar cells that were not pre-treated with Aβ_1-40. _Additional controls such as addition of vehicle (aCMF) and Aβ_1-40 _were also used. For this co-focused K^+ ^and Ca^2+ ^microelectrodes were positioned ~5 μm above the cells and moved by a computer-driven hydraulic micromanipulator by 50 μm away from the cells and back to the primary position with 0.05 Hz frequency. Data was acquired at a rate of 10 samples/sec and averaged later over 6 second intervals. The ion fluxes were recorded for 5 min prior to the addition of 10 μM of glutamate, and recordings continued for a further 25 min. To achieve the required concentration of glutamate in the measuring chamber, an equal volume of the bath solution with a double concentration of glutamate was added resulting in the final concentrations of glutamate (10 μM). Net ion fluxes (nmol m^-2^s^-1^) were calculated using planar geometry of diffusion equations.

### Statistical analyses of tissue culture experiments

For each experiment unless otherwise stated, a minimum of four wells from at least three separate cultures (derived from different animals), were used for quantification. Statistical analysis was completed using SPSS 16.0 (SPSS). When data was unequally distributed, data was transformed so that the residuals were approximately normally distributed. Statistical significance was calculated using One-Way ANOVA with Tukey's Post Hoc Test and Student's *t*-test.

## Abbreviations

Aβ: beta amyloid; AD: Alzheimer's disease; APP: β-amyloid precursor peptide; DIV: days *in vitro*; MIFE: microelectrode ion flux measuring technique.

## Competing interests

The authors declare that they have no competing interests.

## Authors' contributions

LS carried out MIFE experiments, analysed and interpreted data, and drafted the manuscript. CH carried out the survival experiments and participated in data analyses. AKW participated in the design of the study, participated in its coordination and contributed to the preparation of the manuscript. RSC conceived the study, participated in overall direction of the study and preparation of the manuscript. All authors have read and approved the final manuscript.

## Supplementary Material

Additional file 1 **Figure S1. Effects of acute application of Aβ_1-40 _on net K^+ ^and Ca^2+ ^fluxes**. We tested whether acute application of Aβ_1-40 _to 14 DIV cortical neurons affected magnitudes of K^+ ^and Ca^2+ ^fluxes. Net fluxes of K^+ ^(**A**) and Ca^2+ ^(**B**) were recorded for 5 min (-5 to 0 min) followed by acute application of 1 μM Aβ_1-40 _(0 to 10 min) and 40 μM Aβ_1-40 _(final concentration, 10 to 25 min) to the bath. Neither concentration caused changes in net ion fluxes measured during the time course tested suggesting that prolonged treatment with Aβ_1-40 _is required to trigger disturbances in ion homeostasis. Error bars are SEM (n = 4). Click here for file

Additional file 2 **Figure S2. Effects of experimental solution application to the measuring chamber on ion fluxes**. An additional control was made to test a possible effect of solution disturbances in the measuring chamber on ion fluxes. Neurons at 14 DIV were used. Net K^+ ^(**A**) and Ca^2+ ^(**B**) fluxes were recorded continuously for 5 min (-5 to 0 min) and after vehicle (aCSF) application to the bath (0 to 10 min) with data acquired at a rate of 10 samples/sec and averaged over every 6 sec. Vehicle was applied at zero time as indicated by an arrow. No changes in K^+ ^and Ca^2+ ^fluxes were observed thus validating the approach used. Error bars are SEM (n = 4). Click here for file
