# Supplementary material for: Prolonged Aβ treatment leads to impairment in the ability of primary cortical neurons to maintain K+ and Ca2+ homeostasis
Source: Mol Neurodegener. 2010 Aug 13;5:30. doi: 10.1186/1750-1326-5-30 (PMC2927593; doi:10.1186/1750-1326-5-30)
Supplement: Additional file 1 — Figure S1. Effects of acute application of Aβ1-40 on net K+ and Ca2+ fluxes. We tested whether acute application of Aβ1-40 to 14 DIV cortical neurons affected magnitudes of K+ and Ca2+ fluxes. Net fluxes of K+ (A) and Ca2+ (B) were recorded for 5 min (-5 to 0 min) followed by acute application of 1 μM Aβ1-40 (0 to 10 min) and 40 μM Aβ1-40 (final concentration, 10 to 25 min) to the bath. Neither concentration caused changes in net ion fluxes measured during the time course tested suggesting that prolonged treatment with Aβ1-40 is required to trigger disturbances in ion homeostasis. Error bars are SEM (n = 4). [file 1750-1326-5-30-S1.DOC]

**Additional Figure S1**

Efflux Influx

**A**

**B**

**K^+^**

**Control**

**+ 1 μM Aβ**

**0 Aβ 1 μM Aβ**

Efflux Influx

**+ 1 μM Aβ**

**Ca^2+^**

**Control**
