# Supplementary material for: Prolonged Aβ treatment leads to impairment in the ability of primary cortical neurons to maintain K+ and Ca2+ homeostasis
Source: Mol Neurodegener. 2010 Aug 13;5:30. doi: 10.1186/1750-1326-5-30 (PMC2927593; doi:10.1186/1750-1326-5-30)
Supplement: Additional file 2 — Figure S2. Effects of experimental solution application to the measuring chamber on ion fluxes. An additional control was made to test a possible effect of solution disturbances in the measuring chamber on ion fluxes. Neurons at 14 DIV were used. Net K+ (A) and Ca2+ (B) fluxes were recorded continuously for 5 min (-5 to 0 min) and after vehicle (aCSF) application to the bath (0 to 10 min) with data acquired at a rate of 10 samples/sec and averaged over every 6 sec. Vehicle was applied at zero time as indicated by an arrow. No changes in K+ and Ca2+ fluxes were observed thus validating the approach used. Error bars are SEM (n = 4). [file 1750-1326-5-30-S2.DOC]

**Additional Figure S2**

**A**

**B**

Efflux Influx

**+aCMF**

**K^+^**

**Control**

Efflux Influx

**+aCMF**

**Ca^2+^**

**Control**
